# Validation of a dynamic 4D microsurgical bypass simulator for training and teaching microvascular anastomosis techniques with blood flow and fluorescence imaging

**DOI:** 10.1016/j.wnsx.2024.100396

**Published:** 2024-09-21

**Authors:** Hanne Eline R. Vanluchene, David Bervini, Ross Straughan, Samuel Maina, Fredrick J. Joseph

**Affiliations:** a ARTORG center for Biomedical Engineering Research, University of Bern, Bern, Switzerland; b Department of Neurosurgery, Inselspital, Bern University Hospital, University of Bern, Bern, Switzerland

**Keywords:** Cerebral bypass, Microvascular anastomosis, Neurosurgical training, Surgical simulation

## Abstract

**Objective:**

Microvascular anastomosis is challenging, and training surgeons to develop and maintain skills is imperative. Current training models either miss the simulation of the surgical workflow, lack 3D key-hole space, need ethical approval, require special preparation, or lack realism. To circumvent these issues, this study describes the use of a mixed reality 3D printed model with integrated blood flow for training cerebral anastomosis and assesses its validity.

**Methods:**

Different-sized 3D-printed artificial micro artery models in a 3D brain space with a keyhole opening were used. The model was connected to a 4D simulator to induce pulsatile blood flow. Neurosurgical microscopes and exoscopes were used for visualization. Nine participants (n = 6 board-certified cerebrovascular neurosurgeons; n = 3 in-training) participated in the study and practiced anastomosis techniques with the simulator. Two senior, experienced vascular neurosurgeons mentored live teaching activity on the simulator. Participants completed a survey to score the face and content validity of the simulation on a 5-point Likert scale. Simulation time and anastomosis score differences between in-training and board-certified participants were compared for construct validity.

**Results:**

Participants scored the simulation difficulty similar to actual surgery, proving face validity. All participants agreed that practice on the provided simulator models would improve bypass techniques (*μ* = 4.67/5 ± 0.47) and instrument handling (*μ* = 4.56/5 ± 0.68). Board-certified participants had better anastomosis scores than in-training participants (non-significant difference).

**Conclusions:**

The 4D simulator and the high-fidelity artificial 3D printed model effectively simulated actual bypass surgery in a key-hole fashion with blood flow abilities. Limited resources and preparation time are needed for the training setup. The model provides benefits in learning and maintaining anastomosis skills and allows for easy adaptation to different microanatomical scenarios.

## Abbreviation list

ICG: Indocyanine green
MVA: Microvascular anastomosis
STA: Superficial Temporal Artery

## 1 Introduction

Microvascular anastomosis (MVA) is a surgically challenging task. It is regarded as one of the most difficult skills to acquire in various microsurgical fields, namely neurosurgery, plastic surgery, maxillofacial reconstruction, cardiovascular surgery and lymphatic surgery.^1^, ^2^, ^3^, ^4^, ^5^, ^6^, ^7^ For the cerebrovascular field, the complexity is even higher due to evolving deep key-hole approaches, resulting in narrow 3D operative corridors compared to the other specialties. Furthermore, surrounding delicate organs at risk of tissue damage, makes it a demanding and highly specialised procedure in clinical practice.^8^, ^9^, ^10^ Due to advancements in endovascular surgery, the number of microsurgical bypass procedures for cerebral revascularisation is decreasing.^11^^,^^12^ However, microvascular anastomosis in neurosurgery is still essential for several surgeries; complex cerebral aneurysms, moyamoya disease, revascularization on brachiocephalic and carotid arteries, and during resection of brain tumors that obstruct cerebral arteries.^2^^,^^13^^,^^14^ Hence, it is essential that these skills are retained by neurosurgeons for future procedures. Therefore training to develop and maintain the required surgical skills is necessary,^2^^,^^10^^,^^13^^,^^15^ but doing this through incidental surgeries is challenging.^2^^,^^10^ MVA teaching and learning require significant individual commitment and should be undertaken within an appropriate laboratory environment.^9^^,^^10^^,^^16^ To be an effective teaching aid, an MVA training model must be highly realistic and effective in improving manual dexterity, coordination, speed, agility, flexibility, reaction time, whilst reproducing the entire microsurgical workflow. In addition, the training setup should provide the possibility to train cognitive abilities, including synchronized hand-eye coordination, motor skills, and mental strength.^16^, ^17^, ^18^, ^19^

Training on living rats is one of the most used MVA training methods.^10^^,^^20^ It provides high accuracy in simulating the surgical workflow, closely resembling the human microvasculature.^2^^,^^20^ In addition, natural blood flow and patency checks lead to a realistic physiological environment with natural coagulation in place.^2^^,^^9^ However, using living animal models requires specific laboratory conditions, ethical committee approval, and perpetual training which can be challenging.^2^^,^^3^^,^^21^ The simplest existing models are dry models, such as silicone tubes. These are inexpensive, but they lack true resemblance of the 3D anatomy. Mimicking the vessel structure has also been done in wet models, such as preserved rat vessels, chicken or turkey arteries, and human or bovine placentas.^13^^,^^21^, ^22^, ^23^, ^24^ However, these inexpensive wet training modalities are less available than synthetic materials, cannot simulate the physiological properties, require specific preservation maintenance of specimens that can only be preserved for a limited time, and are only widely available in some training centres and few also report unavailability due to socio-cultural and regional practices.^10^^,^^15^ In addition, these dry and wet models do not replicate the depth of the operative corridors nor the limited vascular mobility.^2^^,^^25^ Therefore, using ex-vivo specimens better simulates the surgical workflow. However, human cadavers have other disadvantages, such as long-term sourcing, limited access, limited perfusion, and high resources in preparation.^10^^,^^15^^,^^25^

To address the aforementioned problems, and given the increasing trend towards key-hole surgical procedures, training more often and in advanced simulation models is a solution to develop and maintain the necessary skills required.^26^, ^27^, ^28^ These models should include a simulation of the blood flow, a complete surgical workflow of the key-hole approach and an anatomically representative three-dimensional training space.^2^^,^^16^^,^^25^^,^^28^, ^29^, ^30^ Therefore, we used a substitutive model for training intracranial and extracranial bypass techniques with synthetic materials to reduce animal use, create a reproducible model, and refine microsurgical skills. We used MVA training models developed from the most advanced additive 3D printing technologies to practice micro anastomosis in a 3D space coupled with a 4D mixed reality simulator with pulsating blood flow.

## 2 Materials and methods

**Study participants:** The study included nine participants. Three were in training with three to five years of completed neurosurgical training and had not yet assisted in a bypass surgery. The other six participants were already board-certified neurosurgeons; five had been board-certified for four to six years and had never performed a bypass surgery as the primary surgeon but had assisted in the surgery. The remaining neurosurgeon had been board-certified for 12 years and completed 25–50 bypasses as the leading surgeon.

**Fig. 1:**
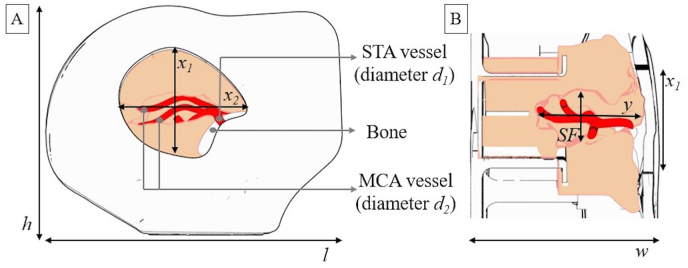
Illustration of exchangeable bypass training model (A. Front view, B. Cross-sectional view), with a realistic representation of the Sylvian fissure width (SF): *l* = 68 mm, *h* = 55 mm, w = 45 mm, x_1_ = 20 mm, x_2_ = 30 mm, y = 25 mm, SF = 7.5 mm, d_1_ = 1.0 mm, d_2_ = 0.8 mm.

**Fig. 2:**
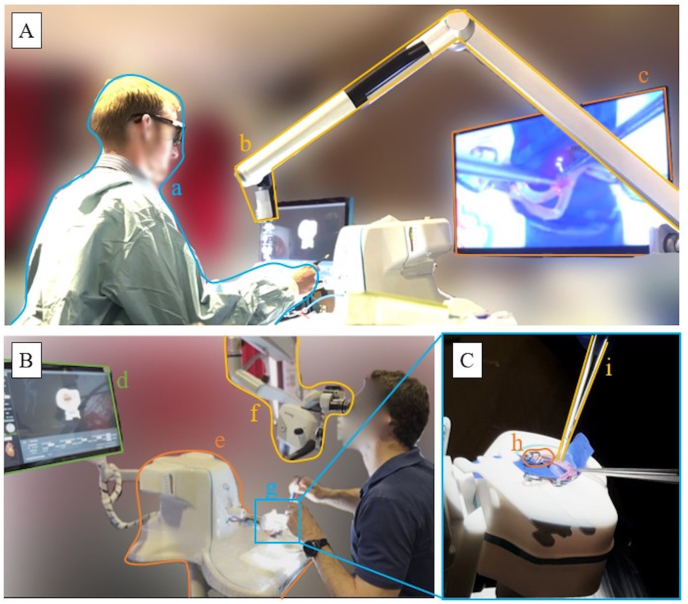
Simulated operation theatre setup in a hands-on workshop setting: A. Simulator set-up with exoscope. B. Simulator set-up with a microscope. C. Anastomosis model with instruments from standard bypass set. (a: participant with exoscope 3D glasses, b: exoscope, c: exoscope display placed 2 m from the participant, d: simulator software display, e: simulator, f: microscope, g: microvascular anastomosis model, h: bypass clips, i: bypass instruments).

**Simulator and models:** The commercially available 4D microsurgical simulator (SurgTrain^TM^, SurgeonsLab AG, Switzerland) was used as a training device.^31^, ^32^, ^33^ It included 3D models with commercially available exchangeable 3D-printed micro arteries with pulsatile blood flow and a brain parenchyma model with more profound sylvian openings embedded, mimicking the 3D spatial difficulties of extra-to intracranial and intra-to intracranial arterial bypass surgery. The micro arteries are patented and included dynamic life-like physiological flow characteristics and allows for injection of Indocyanine green (ICG) from the simulator whenever required. An overview and specifications of the training model can be seen in Fig. 1, where the keyhole approach is illustrated. The training model included one Superficial Temporal Artery (STA) with an inner diameter of 1.0 mm and two MCA recipient vessels with a lumen diameter of 0.8 mm that were 3D printed. The intracranial keyhole depth of the model allows also to recreate deeper bypass techniques such as proximal MCA branches (M1 and M2 segments). This combination allowed for training side-to-side, end-to-end, and end-to-side anastomosis techniques on the same model (mimicking bypass conditions using the simulator). In addition, the simulator with multiple degrees of freedom allowed the participants to position the model in any convenient patient-like head position, allowing them to recreate the actual surgical situation (Fig. 2).

**Microsurgical Instruments and Microscopy:** A standard bypass set (Peter Lazic GmbH, Germany) and a Ethicon wound closure suturing kit containing 9-0 and 10-0 sutures (Johnson & Johnson, NJ, USA) was available for training, including temporary bypass clips, a clip applier, dissectors, micro forceps, micro scissors, and a microneedle holder. An Exoscope and multiple surgical microscopes were used during the simulation training: ORBEYE 4K 3D orbital camera system (Olympus Medical Systems Corp., Japan), Zeiss Opmi Penetro 800, and Zeiss Extaro 300 (Carl Zeiss Surgical GmbH, Germany).

**Mentors and Live Teaching:** Two expert neurosurgeons each with 10–15 years of experience in cerebrovascular neurosurgery were available during the training session to provide feedback during the hands-on session and coach the participants on special bypass techniques using the simulator.

**Fig. 3:**
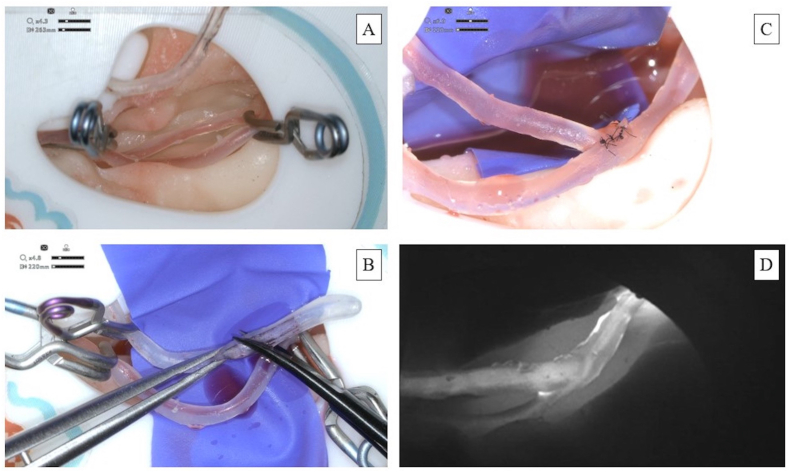
Microsurgical workflow: A. Recipient clamping and preparation. B. Donor preparation C. Anastomosis. D. Vessel opening and ICG inspection.

**Study design:** A complete simulated operation theatre setup was available for the participants, including nine training models with individual simulators, microscopes, and surgical instruments (Fig. 2). All the participants trained in parallel for half a day in a hands-on simulation workshop training setting. The integrated simulator software allowed trainees to follow different microsurgical steps, as represented in Fig. 3. The software was also able to track their performance in terms of the operation time and attempts in each stage of bypass training. The participants started with donor preparation, recipient preparation, and clamping. Subsequently, the anastomosis itself was performed, and, if successfully completed, the vessels opened with an ICG inspection mimicking clinical procedures.^34^ No time restriction was given during the simulated bypass surgery training. After the simulation, participants were asked to complete a survey to provide feedback on their experience.

**Survey instrument:** Face and content validity were investigated using Barrow's Bypass Participant Survey^13^^,^^25^ with a 5-point Likert scale, with the intention to identify the translatable benefits to real surgery. Four questions focused on face validity (representation of actual task, difficulty of procedure, and success in accomplishing task), and three on content validity (model ability to improve bypass technique, handling of instruments and technique when applied to patients).

**Training assessment:** The bypass procedure was recorded under the microscope during most training sessions. The recording duration was used to estimate how long the participants trained on the model. The microscope recordings were also used to score the quality of the anastomosis as shown in Table 1^16^ which is used as a construct validity assessment when comparing the in-training and board-certified participants.

**Table 1:** Anastomosis score scale^16^.

**Score**	**Definition**
1	Total occlusion
2	High leakage
3	Low leakage, Significant stenosis
4	No leakage, significant stenosis
5	Medium leakage, significant stenosis
6	Low leakage, non-significant stenosis
7	No leakage, non-significant stenosis

**Statistical analysis and study outcomes:** Survey responses were coded and double-checked for accuracy. All data was processed using Python. The survey responses were analyzed by visual interpretation based on boxplots. The microscope time and anastomosis score were compared between in-training and board-certified participants. To check if the difference is significant, a standard independent two-sample t-test was used when the two groups have similar variances; if not, a Welch's t-test was used.

## 3 Results

**Fig. 4:**
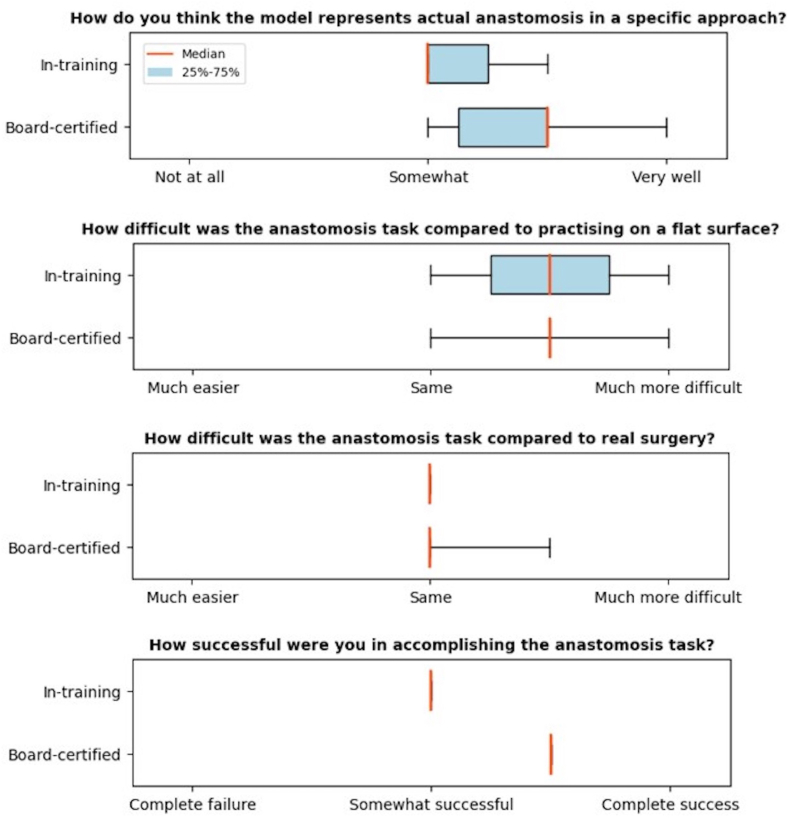
Plots show the outcomes of the face validity.

**Face validity:** The participants scored the ability of the simulation to represent actual microsurgical conditions from ‘somewhat’ to ‘very well’ (score 3–5), with a *μ*_it_ = 3.33 ± 0.47 for the in-training participants and *μ*_bc_ = 3.83 ± 0.69 for the board-certified participants. The participants found that the model was more difficult than practicing on a flat surface with μ = 4.0 ± 0.67 (equating to ‘more difficult’), but the difficulty resembled actual surgery with μ = 3.11 ± 0.34 (∼‘same difficulty’). A successful anastomosis was defined as the anastomosis suturing exhibiting patency. The in-training participants scored their task as somewhat successful, and the board-certified participant as successful but not entirely, as they observed some minor bleeding. An overview of the results of face validity can be seen in Fig. 4.

**Fig. 5:**
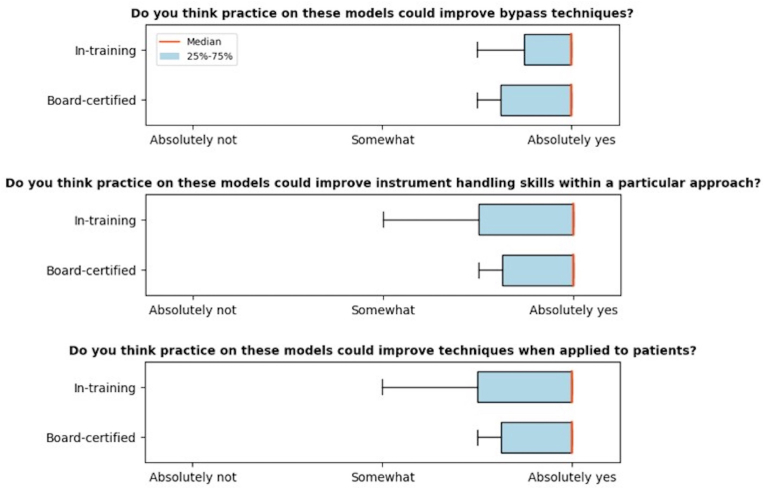
Plots show the outcomes of the content validity.

**Content validity:** Both in-training and board-certified participants agreed that practicing on the models could improve bypass technique (*μ* = 4.67 ± 0.47), instrument handling (*μ* = 4.56 ± 0.68), and techniques when applied to patients (*μ* = 4.56 ± 0.68). The results can be seen in Fig. 5.

**Fig. 6:**
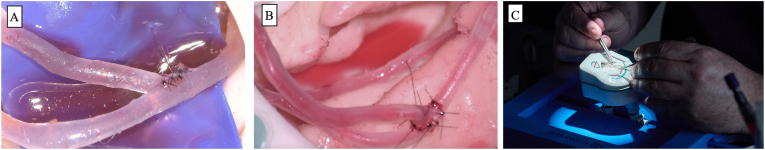
Anastomosis Quality Assessment: Screenshot of microscope video demonstrating. A. High bleeding in anastomosis. B. No bleeding anastomosis from the continuous, pulsatile blood flow from the simulator. C. Simulator recreates narrow keyhole approach.

**Construct validity:** For seven out of nine participants (78 %), a recording of the microscope video was available. The recording time and anastomosis score are summarized in Table 2. An example of anastomosis with high bleeding and without bleeding can be seen in Fig. 6. Participants that were still in training spent more time on the model (*μ*_it_ = 01:16:43 ± 00:18:30) than the board-certified participants (*μ*_bc_ = 01:06:43 ± 00:20:04). The difference is not significant (p = 0.63, two-sample t-test). Board-certified surgeons obtained the highest anastomosis scores. The difference in mean score between in-training participants (*μ*_it_ = 3.0 ± 0.00) and board-certified participants (*μ*_bc_ = 4.0 ± 2.10) is only one point, which is a non-significant difference (p = 0.39, Welch's t-test). This difference is related to the scores of participants 3 and 4. Participant 3 had quite some experience but had total occlusion for the bypass, as the preparation of the donor's vessel was inadequately performed. Participant 4 also scored low as they focused more on the suture technique to create a successful anastomosis after not correctly clamping the vessels securely, however they avoided major bleeding.

**Table 2:** Training time and anastomosis score.

**Participant**	**Experience**	**Training time**	**Anastomosis score (7 maximum)**
1	In-training (3 years)	Not available	Not available
2	In-training (4 years)	00:58:12	3
3	In-training (5 years)	01:35:13	3
4	Board-Certified (4 years)	01:32:59	5
5	Board-Certified (5 years)	Not available	Not available
6	Board-Certified (6 years)	00:46:32	2
7	Board-Certified (6 years)	01:29:17	1
8	Board-Certified (6 years)	00:52:07	6
9	Board-Certified (12 years)	00:52:41	6

## 4 Discussion

State-of-the-art training modalities for bypass techniques either do not realistically simulate the surgical workflow, lack 3D key-hole space, need ethical approval, require specialized training model preparation or require specially designed lab equipment. Therefore, a new training model was used for complex cerebrovascular anastomosis in a hands-on workshop setting to circumvent these problems. The training model incorporated the key-hole approach with anatomically deep micro arteries and blood flow, allowing the participants to train the complete microsurgical workflow. Only one size of arteries was used for the simplicity of the study. Nonetheless, the model facilitates different complexity levels by adapting the size of the arteries, allowing the practice of grafting vessels with large diameters. Bypass integrity was validated by direct blood and ICG injection into the adjacent vessel segments through the simulator software, which also allowed recording and quantifying the performance in a realistic situation alongside the mentor's feedback. A survey was used to assess the model. The answers showed high face and content validity by both in-training and board-certified neurosurgeons. The model provides an accurate representation of an actual anastomosis, and the difficulty level is close to real surgery. Participants believed practicing on the provided simulation model will improve bypass technique and instrument handling. For construct validity, microscope time and anastomosis score were compared between the in-training and board-certified neurosurgeons. The overall performance of board-certified participants was better as the in-training participants spent more time on the models and had lower anastomosis scores. However, the differences were not statistically significant because most of board-certified participants never performed microvascular anastomosis in real-life. Offering clearer instructions to participants on the surgical procedure could potentially improve the performance outcomes in the in-training group and lead to comparable outcomes to the board-certified neurosurgeons. Moreover, board-certified neurosurgeons could improve, reaching the level of cerebrovascular neurosurgeon experience. It provides an effective tool for bypass surgeons to maintain consistency and keep track on their performance.

During the training session, the teaching possibilities of the simulator were shown, and close mentor interaction and nearby assistance with the models were proven. Participants reported getting tired throughout the simulated procedure and experienced difficulty handling the tiny needles and 10-0 sutures with the blood flow. Younger, in-training participants could not continue to suture completely despite bleeding and had incomplete suturing. Some participants reattempted, out of the study scope, after a break with new training models and achieved patency and good feeding from the donors without bleeding. This highly suggests the simulation replicated realistic behavior and showcased the risks involved in real surgery, like putting the participant under stress, building tension with the 3-dimensional aspects of the microanatomy, thereby allowing participants to gain mental strength. Additionally, participants could train in other necessary microsurgical skills, such as dexterity, robustness, steadiness, posture, and handling techniques for different microscopes and exoscope.^35^ The possibility of using different visualization modalities showed the simulator's ability to be used even with the most sophisticated infrastructure, complementing the immersive training setup. Together with the real microsurgical tools and the visualization modalities, the simulator enables training in an environment with the correct ergonomic position.

A study conducted by Vasankari, Hafez et al 2024 utilized an older iteration of the SurgTrain^TM^, 4D Neurosurgical Simulator.^36^ A potential limitation of the study performed by Vasankari, Hafez et al 2024 is that they did not have participiants with real expereince in by-pass surgery. With our board certified participaints, we observed that their average anastomosis score was better, as would be expected, reflecting the realism of the simulator with past training and expereince being relevant even when suturing with synthetic vessels. As our results conform with the observations Vasankari, Hafez et al 2024 indepdently made, there is more statistical validity to the benefits of the training device. Our manufacturing process allows for the various parameters shown in Fig. 1 (x_1_, x_2_, y_1_, d_1_ and d_2_) to be adjusted thereby recreating different anatomies. Although Vasankari, Hafez et al chose models that had more superficial vessels, our models had anatomies with deeper vasculature showcasing its ability to recreate a variety of anatomies and their associated complexity. Furthermore, Vasankari, Hafez et al noted some inconsistent in the size of the vessel walls which have since been ratified through alterations in the manufacturing process, thus making the difficulty in performing the anastomoses much more consistent.

A review of the currently available literature was conducted using the following terms in PubMed: "microvascular anastomosis" AND ("simulation" OR "model" OR "training") AND "neurosurgery". The search demonstrated relative scarcity of studies evaluating 3D printed models and simulators for training in microvascular anastomosis skills.^36^ Most of the remaining studies refer to animal models or ex-vivo models of bovine placenta, reinforcing the need for a more reproducible and more viable simulation and training model.^37^, ^38^, ^39^

**Strengths:** The simulator offers a number of strengths. Firstly, the simulator allows for each stage of the workflow to be automatically recorded, thereby helping to identify potential inefficiencies that participants have. In addition to mentors reviewing the participants in person, the recording of the microscopic videos enables experts to score the anastomosis for various metrics such as suturing quality. Unlike many other simulators, as the model incorporates ICG marker, clinical procedures can be more closely replicated. Different anastomosis techniques can be performed on the model due to the multiple integrated vessels. Unlike ex-vivo specimens, the micro-arteries are reproducible, and the training model can be easily replaced after multiple trainings. The synthetic simulation can be performed in a simple workshop setting with limited resources: no time-consuming preparation nor specific equipment, such as refrigerators or a specially equipped lab, are required, allowing for short-span training sessions despite regional and infrastructure limitations. Therefore, the 4D microsurgical simulator is a valid substitution for animal and other in vivo training models as it is valuable, realistic, robust, and reusable. It can be introduced as a part of the neurosurgical education armamentarium to guarantee high training standards for present and future generations of surgeons performing microsurgical procedures.

For effective training, a simulator must be readily and easily accessible. The 3D printing manufacturing processes that we utilize facilitates scaling up manufacturing significantly, thereby reducing costs of models to a few hundred dollars. Other simulation models are often made by hand and thus require extensive labour, thereby requiring an often-overlooked monetary cost associated with salaries of technical staff.^40^ Further to this point, manually creating the models not only reduces the consistency of the anatomy, especially those made from real tissue, but consequently also the reproducibility of simulated procedures, reducing the validity of comparisons made between the procedures made by various participants.^13^^,^^21^^,^^24^^,^^41^ Cadavers used for training often requires specially designed and licensed rooms that are not otherwise needed for synthetic simulators. Unlike manual techniques, 3D printing allows for deep anatomical structures such as proximal branches of the MCA (shown in Fig. 1) to be incorporated.

**Limitations:** We acknowledge some study limitations. The vessels were rigid and floated around in the model while suturing. The Sylvian fissure was open, and no dissections aspects were involved. More investigation regarding the structure of the vessels is needed. This could include a representation of the different layers of a natural vascular wall to make the simulation even more realistic. Another disadvantage of the model is that bleeding cannot be controlled at times; realistically, no blood clotting is possible. The study itself also had a few limitations. First, there were only a limited number of participants and no information was retrieved on which previous training modalities they practiced on for bypass. Secondly, microscope videos were used to assess the anastomosis score and simulation duration, as some participants did not activate the tracking software correctly. Finally, the study is also subject to incomplete data availability, as videos were only available for some participants, leading to missing information for two participants.

**Future directions:** Using the simulator and the models during mock-up sessions in a completely simulated surgical environment, including the surgical team, would allow for training teamwork and mastering microvascular surgical techniques. The models and simulator could even train autonomous devices for microsurgical procedures in a controlled, repetitive fashion. Improvement in the micro arterial layers and inclusion of dissection aspects can provide additional values that need to be added in future studies. Integration of this simulator in training curricula or off-the-job training would translate to surgeons being well-trained to handle complications, surgeons maintain surgical consistency for less common procedures, improved patient safety, and reduced surgical morbidity and associated costs. Further research with this and similar simulators should be investigated further to trends and observations statistically stronger with a larger number of participants conducted at different research institutes.

## 5 Conclusion

The high-fidelity simulator and the artificial 3D printed model efficiently simulated actual bypass surgery with a deep and narrow key-hole approach and with blood flow abilities. The model can serve as a valid substitution for animal training models and is beneficial for learning and maintaining anastomosis skills. The simulator can be used as a stand-alone mentorship tool with no maintenance, limited resources, and no contingencies. The artificial training model will improve confidence, develop competence, translate knowledge, and provide better performance experience in an actual clinical case, demonstrating the perpetual training ability of the simulator.

## Funding

This research did not receive any specific grant from funding agencies in the public, commercial, or not-for-profit sectors.

## CRediT authorship contribution statement

**Hanne Eline R. Vanluchene:** Writing – review & editing, Writing – original draft, Visualization, Validation, Project administration, Methodology, Investigation, Formal analysis, Conceptualization. **David Bervini:** Conceptualization, Methodology, Writing – review & editing. **Ross Straughan:** Formal analysis, Writing – review & editing. **Samuel Maina:** Writing – review & editing, Methodology, Data curation. **Fredrick J. Joseph:** Writing – review & editing, Supervision, Methodology, Conceptualization.

## Declaration of competing interest

The authors declare the following financial interests/personal relationships which may be considered as potential competing interests: Fredrick J. Joseph and David Bervini report a relationship with SurgeonsLab AG that includes: board membership and equity or stocks.
